# Muscle and Tendon Stiffness of the Lower Limb of Professional Adolescent Soccer Athletes Measured Using Shear Wave Elastography

**DOI:** 10.3390/diagnostics12102453

**Published:** 2022-10-11

**Authors:** Claudia Römer, Julia Czupajllo, Enrico Zessin, Thomas Fischer, Bernd Wolfarth, Markus Herbert Lerchbaumer

**Affiliations:** 1 Department of Sports Medicine, Charité-Universitätsmedizin Berlin, Corporate Member of Freie Universität Berlin, Humboldt-Universität zu Berlin, 10117 Berlin, Germany; 2 Department of Radiology, Charité-Universitätsmedizin Berlin, Corporate Member of Freie Universität Berlin, Humboldt-Universität zu Berlin, 10117 Berlin, Germany

**Keywords:** shear wave elastography, SWE, tendon, muscle, ultrasound, soccer, adolescence

## Abstract

Background: While adolescents have specific risk factors for acute and chronic injury, there is a lack of preventive medicine algorithms for this vulnerable group. Shear wave elastography (SWE) is currently mainly used for assessing muscle and tendon stiffness in adult athletes and can diagnose tissue pathologies such as tendinopathy. The aim was to investigate differences in quadriceps tendon and muscle stiffness between adolescent and adult professional soccer players using SWE and identify lateral imbalances in order to improve the knowledge of preventive medicine algorithms for professional adolescent athletes. Methods: Standardized SWE examinations of both lower limb tendons and muscles (the quadriceps tendon (QT) and the vastus medialis (VM) muscle) in the longitudinal plane and relaxed tendon position were performed in 13 healthy adolescent soccer athletes (13–17 years), and a control group of 19 healthy adult professional soccer athletes (18–29 years). Results: Adolescent soccer players had lower stiffness values for both the quadriceps tendon (3.11 m/s vs. 3.25 m/s) and the vastus medialis muscle (1.67 m/s vs. 1.71 m/s) than adult athletes. Moreover, QT stiffness in adolescent soccer players was significantly lower on the right side (QT: adult 3.50 m/s (2.73–4.56) vs. adolescent 2.90 m/s (2.61–3.12); *p* = 0.031). Analysis of the lateral differences revealed softer QT and VM tissue on the right side in over two-thirds of adolescent soccer athletes. Over two-thirds of adults had stiffer QT and VM tissue on the right side. Conclusion: In adolescent soccer players, the stiffness of the QT and VM muscle measured by SWE is lower in the right leg. SWE of the musculoskeletal system may thus become a relevant diagnostic tool to detect early lateral imbalances as a main risk factor for injury and may thus contribute to the prevention of acute and chronic injury prevention in adolescent athletes.

## 1. Introduction

Adolescent athletes specialize early during their sports career [1]. Not only specialization in younger years, which results in monotonic locomotion, but also adolescence by itself may be a risk factor for a higher prevalence of injury in young athletes [1,2,3]. Risk factors for injuries in adolescent athletes include a general high training load, lateral imbalances, hormonal and musculoskeletal changes, and lower neuronal control [1,4]. Injuries of the lower limb are dominant complications in professional athletes, especially in soccer players [5]. Furthermore, athletes with a previous knee injury have a higher risk for re-injury [6].

In musculoskeletal disorders and especially sports medicine, there is an increasing interest in the quantification of elastic properties of skeletal muscles and tendons to improve the understanding of alterations in skeletal disorders on resulting pain or injuries in athletes. Furthermore, the goal is a general understanding of the underlying mechanisms and their relationships between these measured parameters and musculoskeletal pathologies. Muscle and tendon stiffness of the lower limb can be measured by shear wave elastography (SWE) and can be used as parameters to identify pathologies such as rupture or tendinopathy [7,8,9,10]. Today, most studies concentrate on an assessment of the Achilles tendon (AT) or patellar tendon (PT) due to common localizations of overuse injuries. [9,11] It is already known that healthy athletes demonstrate significantly higher SWE-values of the Achilles tendons compared to healthy nonathletic participants (e.g., higher tendon stiffness), possibly as a result of repeated long-term training [11]. Interestingly, Dirrichs et al. denoted no difference of Achilles tendon stiffness measured by SWE between the left and right leg in athletes and non-athletic participants [11].

Several studies have shown that tissue stiffness measured by SWE is a suitable parameter for assessing knee tissue such as the quadriceps tendon (QT) and quadriceps muscle (QM) [12,13,14]. SWE of different quadriceps compartments revealed similar changes during passive stretching [15]. An investigation in runners suggested that a decrease in quadriceps muscle stiffness after long distance running might be a sign of beginning quadriceps swelling due to overuse or inflammation [14]. The examination of the quadriceps muscle by ultrasound (US) showed good results in terms of reproducibility, strength, injury, or muscle thickness [16,17]. However, there are fewer studies assessing both the quadriceps tendon and muscle stiffness by using SWE [13,18,19,20,21]. For comparability and the consistency of results, it is important to perform SWE of the quadriceps tendon and muscle stiffness at a standardized knee angle and with a relaxed quadriceps muscle [19,22].

Professional soccer athletes have one of the highest injury prevalence in sports, especially for knee injuries [23,24]. Quadriceps tendon stiffness in professional athletes was lower in comparison to healthy sedentary individuals [25], which can be a risk factor for tendinopathy [8]. Data on quadriceps muscle and tendon stiffness, pathology-related changes, and lateral differences are still lacking for professional adolescent athletes, who have a higher risk of injury than adult athletes [1,2]. To address this research gap, we conducted a prospective study to investigate quadriceps muscle and tendon stiffness using shear wave elastography in adolescent and adult professional soccer athletes.

The aim of this study is to investigate the differences in quadriceps tendon and muscle stiffness of adolescent professional soccer athletes in comparison to adult professional soccer athletes using SWE and to identify lateral imbalances in order to develop preventive medicine algorithms for professional adolescent athletes.

## 2. Materials and Methods

### 2.1. Study Population

The prospective study included 13 healthy adolescent professional soccer athletes and 19 adult professional soccer athletes as controls. Professional football athletes were examined as part of the regular pre-season examination in 2020 at the Sports Medicine Institute of Charité University Medicine Berlin. Written informed consent to participate in the study was given after medical education of a sports medicine physician. The inclusion criteria were: (I) age of 13–17 years for adolescent athletes and ≥18 years for adult athletes, (II) healthy adolescent and adult professional soccer athletes, (III) no acute (>6 months) musculoskeletal, rheumatic, or vascular comorbidities and no previous injuries of the quadriceps muscle or tendon, (IV) comparable training load, and (V) written informed consent to participate in the study. The study was conducted in accordance with the Declaration of Helsinki and was approved by the local ethics committee of Charité University Medicine Berlin (ethical vote number EA2/162/19).

As training load does affect muscle and tendon stiffness, adolescent athletes with mostly more than ten hours training per week were recruited. Especially in the adolescent group, there was a high drop-out rate due to comparable training load in the adult group. In the adult group, there was a drop-out rate of six athletes due to training load. This needs to be considered as a limitation factor of this study. The baseline characteristics of study participants were obtained using a predefined questionnaire. On the day of the SWE examination, no training was performed. All examinations were jointly performed in consensus by a trained sonographer and a highly experienced radiologist specialized in ultrasound imaging using a standardized protocol.

### 2.2. Ultrasound-Based Shear Wave Elastography

The SWE measurements were performed as part of a pre-season examination, including a complete physical examination, an ECG, a lung function test, laboratory diagnostics, and lactate performance diagnostics. All athletes were approved for next season participation. All ultrasound-based shear wave elastography (US-SWE) examinations were performed using a standardized protocol. For the examination of the quadriceps muscle and tendon (mid-portion), the volunteers were positioned in a relaxed supine position with a small roll under the knee (20° flexion). The subjects were asked to stay as relaxed as possible. Prior to the US-SWE, a gray-scale B-mode US was performed in transverse and longitudinal planes to ensure an adequate assessment of the tendons and probe position. All of the examinations were performed using a high-end US system with a 4–10 MHz multifrequency linear array transducer and a center frequency of 7 Mhz (Acuson Sequoia, Siemens Healthineers, Erlangen, Germany). The US-SWE software (Virtual Touch™) allows real-time measurement using Acoustic Radiation Force Impulse (ARFI) imaging technology for the measurement of shear wave speed (SWS). The quantitative measurement is expressed as shear wave velocity (SVW) in meters per second (m/s).

**Figure 1:**
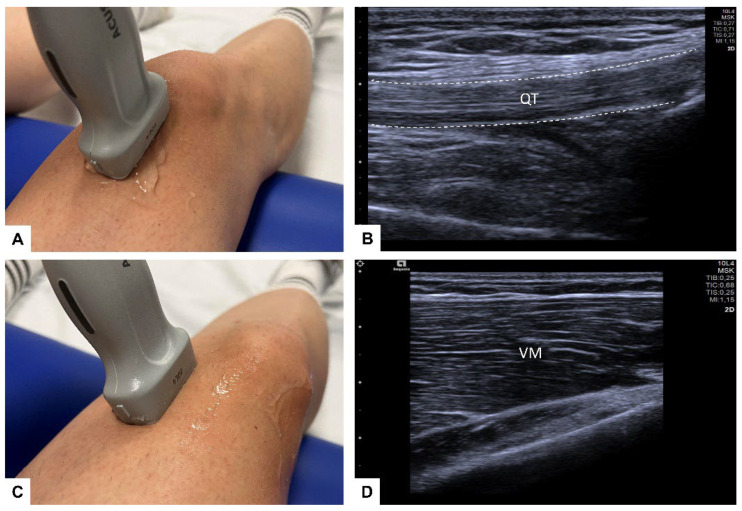
Probe placement and target volume: for SWE, a multifrequency linear transducer was placed in longitudinal plane for accurate assessment of the quadriceps tendon (QT; (**A**,**B**)) and with minimal movement to assess the medial vastus muscle (VM; (**C**,**D**)).

**Figure 2:**
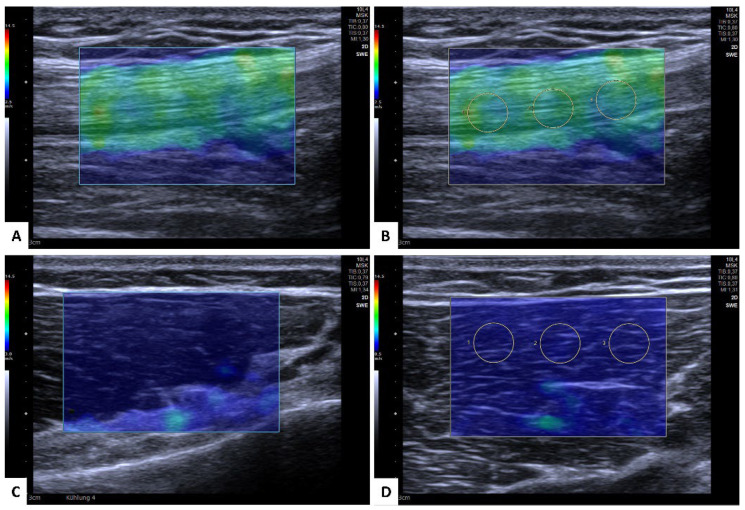
Shear wave elastography of the quadriceps tendon (**A**,**B**) and medial vastus muscle (**C**,**D**) with color-coded map (**A**,**C**) and assessment of shear wave speed using three ROI-measurements per image (**B**,**D**). The color scale on the left side of each image can be used for interpretation of the shear wave speed, ranging from minimum 0 m/s (e.g., very soft = blue) to a maximum of 15 m/s (e.g., red = stiff). In this example, the tendon stiffness is visualized green (resulting in a moderate stiffness around 2–3.5 m/s; Figure 1A,B), while the softer muscle tissue ranged around 1 m/s and is therefore visualized as blue (Figure 1C,D).

The US-SWE examinations were performed in the longitudinal plane to depict each tendon and muscle with the corresponding area of interest in a single image (Figure 1). Using the respective 2D SWE approach, the examiner acquired ten measurements of each tendon and muscle of both legs, corresponding to a total of 1280 consecutive SWE measurements in 3 mm circular regions of interest (ROIs) placed in the center of each target tendon and muscle, avoiding areas of visible artifacts (Figure 2). Thus, the representative tendon and muscle stiffness of each participant is given as the median of 10 measurements and corresponding interquartile range (IQR). Before the ROI placement, the SWS as a surrogate for tissue stiffness was depicted by color-coded SWE mapping (Figure 2A,C). The standardized penetration depth was adapted to each participant for optimal visualization of the tendon and a correct SWE measurement. The gain was not changed to avoid potential effects on the US-SWE results. Special care was taken to avoid inducing pressure on each muscle and tendon while preserving optimal probe coupling during each measurement.

### 2.3. Statistical Analysis

The continuous variables were tested for normal distribution using the Kolmogorov–Smirnov test. The not normally distributed variables are reported as the median and interquartile range (IQR). The categorical variables were compared using Student’s t-test and are reported by their proportion (*n*/N). A two-sided significance level of α = 0.05 was defined as appropriate to indicate statistical significance. All statistical analyses were performed using the SPSS software (IBM Corp., released 2019. IBM SPSS Statistics for Windows, Version 26.0. Armonk, NY, USA: IBM Corp.).

## 3. Results

### 3.1. General Characteristics of the Study Population

A total of 32 professional athletes (adolescent *n* = 13; adult *n* = 19) were examined. All athletes were free of complaints at the time of the measurement. In the adult group, the following conditions were reported: three athletes suffered from anterior cruciate ligament (ACL) rupture and were scheduled for surgery. Three complete and one partial meniscal ruptures were reported. Five athletes suffered from ligament rupture of the upper ankle joint and one athlete reported a fracture of the talocrural joint. Another athlete suffered from a fibula fracture with a rupture of the tibiofibular syndesmosis and one athlete stated a stress fracture of the left foot. In the adolescent group, one athlete reported a tendon rupture of the biceps femoris muscle, one athlete suffered from bilateral Schlatter’s disease, and another athlete reported bilateral pes planus. No other diseases such as diabetes mellitus, fatigue, hyperlipidemia, rheumatic diseases, or malposition of lower limb joints were known. No medications were taken at the time of the examination.

Thirteen adolescent athletes with a mean age of 15.62 [13,14,15,16,17] years and a mean BMI [kg/m^2^] of 21.79 (18.61–24.39) were examined. All subjects in the adolescent group trained for >10 h per week, except for one thirteen- and one fourteen-year-old athlete, who trained 5–10 h per week.

Nineteen adult athletes with a mean age of 20.84 [18,19,20,21,22,23,24,25,26,27,28,29] years and a mean BMI [kg/m^2^] of 23.38 (21.59–26.28) were examined. All adult athletes trained for >10 h per week.

### 3.2. Results of US-SWE in Adolescent and Adult Soccer Athletes

In the total study population (*n* = 32), the quadriceps tendon stiffness values were significantly higher than the stiffness values of the VM. The stiffness values for the quadriceps tendon and vastus medialis muscle were lower in adolescent athletes than in adult athletes. The QT stiffness was significantly lower in adolescent soccer athletes than in adult athletes on the right side (*p* = 0.031), while there was no significant difference on the left side. For the VM in both legs, there were no significant differences between the adolescent and adult athletes. The metric values of all measurements are shown in Table 1.

**Figure 3:**
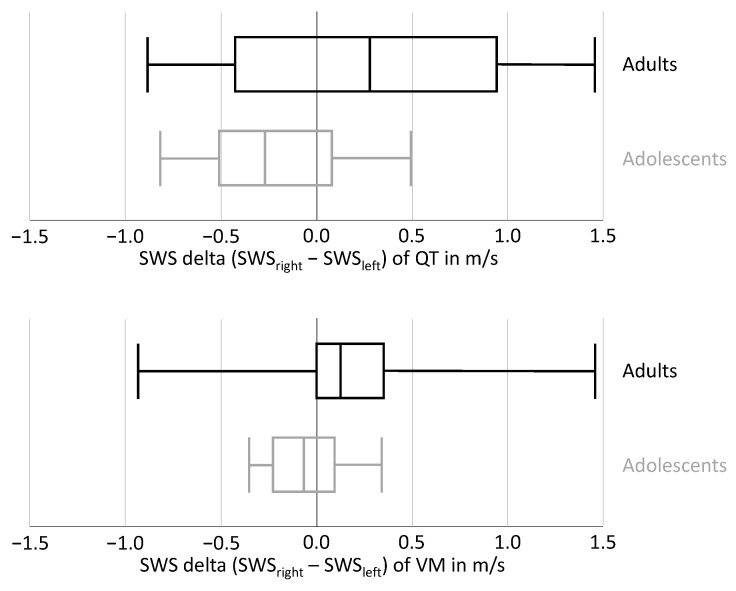
Boxplots of shear wave speed delta (SWS) for adolescent and adult soccer athletes for right and left quadriceps tendon (QT) and medial vastus muscle (MV).

An analysis of the lateral differences (shear wave speed delta for adolescent and adult soccer athletes for right and left) revealed softer QT and VM tissue on the right side in over two-thirds of adolescent soccer athletes (69.23%; 9/13), see Figure 3. Over two-thirds of adults had stiffer QT (31.58%; 13/19) and VM (21.05%; 15/19) tissue on the right side.

## 4. Discussion

Injury prevalence in adolescent athletes is rising, which is attributable to musculoskeletal imbalances due to early sport specialization [1,4]. As an injury is a significant risk factor for re-injury [6], the prevention of injury is of special importance in adolescent athletes. SWE has been shown to be able to detect changes in muscle and tendon stiffness, which can be a sign of injury [8,14]. As knee injuries are predominant in professional soccer athletes [2], our study focused on the lateral differences in stiffness of the lower limb, analyzing QT and VM stiffness in adolescent and adult soccer players.

**Figure 4:**
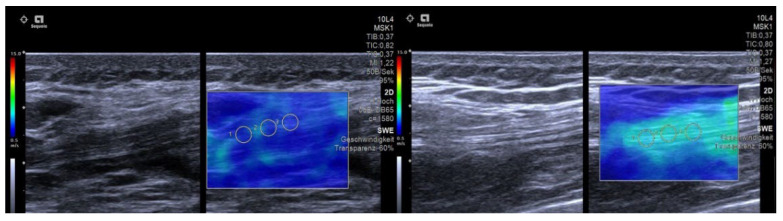
Exemplary images of the right quadriceps tendon in an adolescent soccer athlete (**left**) and an adult athlete (**right**). As depicted by the color-coded map, SWE showed significant higher shear wave velocity in an adult athlete (green, right image) compared to adolescent athlete (left image).

Our results show consistently lower SWE values for adolescent athletes in comparison to adult athletes (Figure 4). This finding may be attributable to a lower muscle mass, resulting in less tendon stiffness in adolescent athletes, who are thus more prone to injury due to greater instability [1,26]. Hormonal status may also play a role and account for the lower SWE values in adolescent athletes in our study [1], while training loads were comparable between the two groups and thus cannot account for the differences. A high training load and an increase in training intensity were described as an important risk factor for injuries in adolescent athletes [4]. The training load of adolescent athletes should be successively increased and compensation training should be included [4,27].

For the quadriceps tendon, a significantly lower stiffness was measured on the right side in our adolescent group. Although approx. 80% of professional soccer athletes are right dominant leg soccer players [28], our data show softer tissue values for the QT and VM on the right side in approx. 70% of adolescent athletes. For adult soccer athletes, our data confirm the prevalence of right leg dominance, with 70% (for the QT) to 80% (for the VM) having stiffer tissue values on the right side. These findings are consistent with a high training load in the adolescent group and might even point to beginning overtraining, as a decrease in tissue stiffness of the quadriceps muscle (vastus medialis, vastus lateralis, and rectus femoris muscle) was reported to be induced by inflammation or muscle swelling [14].

The prediction of injuries using a model based on an orthopedic examination and measurements alone was found to be rather poor [29]. Thus, there is a need to improve injury prediction and develop dedicated approaches for preventive medicine tailored to minimalize acute and chronic injuries in this vulnerable athlete clientele [5,30]. SWE may detect early imbalances in athletes performing asymmetric sports, which was found to be a risk factor for injury in earlier studies and should be investigated further in future studies for more sport types [2,31]. In a prospective study including 462 participants with a standardized concentric and an eccentric isokinetic assessment (preseason and follow-up), the rate of muscle injury of the thigh was significantly higher in participants with untreated strength imbalance compared to participants without lateral imbalance in the preseason [32]. Furthermore, in addition to a higher risk of injury while playing without balanced muscle strength performance, Verrall and colleagues suggested a decrease in playing performance which they investigated in Australian soccer players [33]. In this study, athletes had a significantly lower player performance rating (measured 0–10) immediately upon return to the sport compared to the entire season and compared to the performance ratings derived from the two games prior to injury (both *p* < 0.001). This suggests that injury prevention starts with an accurate preseason assessment. In general, both isokinetic testing and US-SWE assessment can be implemented in a preseason assessment, while intraindividual training control and injury prevention is mandatory for high-end athletes beside sports performance. For non-professional athletes, the implementation of systematic isokinetic testing would probably be too expensive and time consuming with regard to the time allocated to training. Thus, the potential of US-SWE may be a fast and easy parametric assessment that can be used to adapt the training load with a view to minimizing injury in younger athletes. In general, it could be easily implemented during the preseason assessment and as a follow-up tool for intraindividual training control, in both professional and hobby athletes.

### Technical Aspects of US-SWE

Operator dependency remains the major limitation of US and advanced US applications such as SWE, since US needs–beside image interpretation–the acquisition of standardized and representative images done by the investigator. Porta and colleagues reported a -high inter- and intra-observer agreement in the SWE assessment of different compartments of the patellar tendon in healthy participants [34]. The intra-observer agreement was nearly perfect for the experienced investigator and substantial for the unexperienced investigator (e.g., k > 0.61) in the proximal, middle, and distal compartment of the tendon, reaching the highest value for the middle compartment. Since SWE is known as a fast and non-invasive US application, the authors noted a mean time of five minutes for one inexperienced investigator at the beginning of the study, which decreased to two minutes for both experienced and inexperienced investigators. This underlines the fast approach for MSK elastography as an additional tool in multiparametric US. Dickson et al. found a higher reliability of elastography assessment in the patellar tendon compared to the quadriceps tendon in twenty healthy participants [35]. This may be due to the fact that the US assessment of the quadriceps tendon needs more experience, and the visualization of the more superficial patellar tendon is generally associated with a higher image quality.

In terms of muscle stiffness, Tas et al. found a perfect inter-(ICC, 0.95) and intra-observer (ICC, 0.93–0.94) agreement among two investigators for the stiffness measurements with SWE of the rectus femoris muscle. Moreover, the reliability of the measurements on different days was nearly excellent, reaching an ICC of 0.81–0.91 [12]. The point-SWE technique demonstrated as a reliable method for measuring tendon and muscle stiffness in the vastus medialis muscle (*p* = 0.285) and quadriceps tendon (*p* = 0.979) in subjects who had undergone orthopedic surgery [13]. The ICC was excellent for all measured localizations including the vastus medialis muscle (ICC, 0.969) and quadriceps tendon (ICC, 0.995), respectively. The reliability of the measurements shows the possibility of many applications in monitoring stiffness during rehabilitation or as a preventive assessment.

Standardization is the major key in reproducible SWE measurements of intraindividual participants. At the current time, no standardized examination protocol exists for SWE in musculoskeletal applications. Especially, the SWE assessment of the quadriceps tendon and muscle may be influenced by a different knee angle position and quadriceps muscle force. Published by Xu et al., the shear modulus of the rectus femoris muscle was higher than that of the vastus medialis and lateralis muscle if the muscles were stretched over 54° (*p* < 0.01) [15]. The results show that only the superficial heads of the quadriceps muscle generate passive tension almost at the same knee angle (slightly over 40° flexion) during passive knee flexion [15]. Thus, passive knee flexion may hamper not all compartments of the quadriceps muscle. Besides passive stretching, sports activity directly before or days prior to the SWE assessment may influence the quantitative results. It was demonstrated that prolonged and mainly eccentric low-intensity exercises induce changes in quadriceps muscle stiffness, quantified by SWE [14]. The authors observed a significant decrease in shear wave speed and shear wave modulus between the pre and finish sessions during an ultramarathon (*p* < 0.001). Interestingly, a lower but still significant difference in quantitative SWE parameters was also noted between the baseline (prior) and after more than 45 h of recovery (*p* = 0.002). Thus, an investigator who performs quantitative SWE must keep in mind that baseline imaging done in the preseason may be influenced by a higher training load in the days prior to the assessment (for example, in soccer players).

Due to the wider availability of elastography on commercial US systems, there is an increasing number of publications on the topic of US elastography (both SWE and strain elastography) over the last years. The European Society of Radiology formed a consensus statement of US in musculoskeletal applications in 2018, including the discussion on US elastography [36]. Despite the high number of articles reviewed, the authors observed only a limited increase in the evidence level for elastography in a small number of musculoskeletal disorders. They noted an increased level of evidence only for Achilles tendinopathy, lateral and medial epicondylitis, and ultrasound elastography (level of evidence from D to B with an indication grade of 3). For other anatomical regions, such as the quadriceps or patellar tendon, US elastography showed no increase in evidence level or indication grade compared to standard B-Mode US. Beside tendon and muscle applications, the use of US elastography was scored with evidence level B and an indication grade of 1 for soft tissue tumor examination. The main limitation of US elastography may remain to the fact that most published studies are preclinical or feasibility studies and mainly focused on Achilles tendons. However, the growing implementation of elastography in musculoskeletal US should increase the number of studies with the potential to show the impact in clinical practice. For more aspects in muscle and tendon applications, a consensus paper based on newer literature evidence is needed to guide the implementation in clinical practice.

Beside SWE, there are other methods that can be used for the evaluation of tissue stiffness, such as optical coherence elastography. As presented by Liu et. al, the authors obtained the nasal septum cartilage of a pig and used an experimental laser and photodetector setup for the measurement of elastic waves with phase-sensitive optical coherence tomography [37]. In comparison to US, this form of measurement requires tissue extraction and a highly experimental setup, which may be a future perspective with more standardization compared to US (which is known as operator dependent and differs in the US machines of other companies), but not feasible during clinical routine. The power of US-SWE is a direct measurement of tendon or muscle stiffness added to the information of B-Mode US and Doppler US techniques.

Our study has limitations which include a small number of study subjects and the rather young age of the adult control group. This resulted from the inclusion criteria of comparable training loads and methods for professional adolescent and adult soccer athletes. Furthermore, more muscle heads of the quadriceps muscle should be assessed in future studies. Stiffness changes were detected in all muscle heads after intense exercise, as a sign of overtraining [14], thus this could be a better method to investigate the extent of a lateral imbalance in young athletes.

Overall, SWE seems to be a useful tool to assess tissue stiffness not only in adult but also in professional adolescent athletes [11,38]. Individual regular monitoring by SWE in young professional athletes might contribute to reducing the risk of acute and chronic injury by detecting changes in musculoskeletal stiffness and lateral imbalances, as already suggested by earlier studies [7,9]. In addition, SWE-derived stiffness could lead to further preventive measures such as training adaption and compensation training, as well as coach education [3,24,39].

## 5. Conclusions

Adolescent professional soccer athletes show significantly lower stiffness values of the quadriceps tendon than adults, and they have lower stiffness values in the right quadriceps tendon and vastus medialis muscle. Higher tendon stiffness in adult athletes may be a result of a repeated long-term training load. SWE of the musculoskeletal system can be used to detect early lateral imbalances, a major risk factor for injuries in young athletes, and thus may contribute to injury prevention. There is a need to establish preventive medicine algorithms for adolescent athletes to avoid both acute and chronic injuries. SWE has the potential to become a relevant diagnostic tool in this setting.

## Figures and Tables

**Table 1:** Mean shear wave speed (SWS) of the quadriceps tendon and medial vastus muscle in professional adolescent and adult athletes (continuous variables not following a normal distribution given as median and IQR). QT denotes quadriceps tendon; VM, M. vastus medialis.

Tendon and Muscle	SWS Adolescents (m/s) *n* = 13	SWS Adults (m/s) *n*= 19	*p*-Value
QT right	2.90 (2.61–3.12)	3.50 (2.73–4.56)	0.031
QT left	3.11 (2.88–3.53)	3.25 (2.37–3.79)	>0.05
VM right	1.61 (1.36–1.87)	1.88 (1.53–2.27)	>0.05
VM left	1.67 (1.4–1.87)	1.71 (1.37–2.05)	>0.05

## Data Availability

The data presented in this study are available on request from the corresponding author. The data are not publicly available due to data privacy regulations.
